# Do Regions of Increased Inflammation Progress to New White Matter Hyperintensities?: A Longitudinal Positron Emission Tomography-Magnetic Resonance Imaging Study

**DOI:** 10.1161/STROKEAHA.122.039517

**Published:** 2023-01-09

**Authors:** Daniel J. Tozer, Robin B. Brown, Jessica Walsh, Young T. Hong, Guy B. Williams, John T. O’Brien, Franklin I. Aigbirhio, Tim D. Fryer, Hugh S. Markus

**Affiliations:** 1 Stroke Research Group (D.J.T., R.B.B., J.W., H.S.M.), University of Cambridge, United Kingdom.; 2 Wolfson Brain Imaging Center (Y.T.H., G.B.W., F.I.A., T.D.F.), University of Cambridge, United Kingdom.; 3 Department of Clinical Neurosciences, and Department of Psychiatry (J.T.O.B.), University of Cambridge, United Kingdom.

**Keywords:** CADASIL, cerebral small vessel disease, magnetic resonance imaging, positron emission tomography, white matter

## Abstract

Recent studies have demonstrated increased microglial activation using ^11^C-PK11195 positron emission tomography imaging, indicating central nervous system inflammation, in cerebral small vessel disease. However, whether such areas of neuroinflammation progress to tissue damage is uncertain. We determined whether white matter destined to become white matter hyperintensities (WMH) at 1 year had evidence of altered inflammation at baseline.

**Methods:**

Forty subjects with small vessel disease (20 sporadic and 20 cerebral autosomal dominant arteriopathy with subcortical infarcts and leukoencephalopathy) and 20 controls were recruited to this case-control observational study from in- and out-patient clinics at Addenbrooke’s Hospital, Cambridge, UK and imaged at baseline with both ^11^C-PK11195 positron emission tomography and magnetic resonance imaging; and magnetic resonance imaging including diffusion tensor imaging was repeated at 1 year. WMH were segmented at baseline and 1 year, and areas of new lesion identified. Baseline ^11^C-PK11195 binding potential and diffusion tensor imaging parameters in these voxels, and normal appearing white matter, was measured.

**Results:**

Complete positron emission tomography-magnetic resonance imaging data was available for 17 controls, 16 sporadic small vessel disease, and 14 cerebral autosomal dominant arteriopathy with subcortical infarcts and leukoencephalopathy participants. ^11^C-PK11195 binding in voxels destined to become new WMH was lower than in normal appearing white matter, which did not progress to WMH (−0.133[±0.081] versus −0.045 [±0.044]; *P*<0.001). Mean diffusivity was higher and mean fractional anisotropy lower in new WMH voxels than in normal appearing white matter (900 [±80]×10^−6^ versus 1045 [±149]×10^−6^ mm^2^/s and 0.37±0.05 versus 0.29±0.06, both *P*<0.001) consistent with new WMH showing tissue damage on diffusion tensor imaging a year prior to developing into new WMH; similar results were seen across the 3 groups.

**Conclusions:**

White matter tissue destined to develop into new WMH over the subsequent year is associated with both lower neuroinflammation, and white matter ultrastructural damage at baseline. Our results suggest that this tissue is already damaged 1 year prior to lesion formation. This may reflect that the role of neuroinflammation in the lesion development process occurs at an early stage, although more studies over a longer period would be needed to investigate this further.

Cerebral small vessel disease (SVD) causes a quarter of all strokes (lacunar strokes),^1^ and is the most common pathology underlying vascular cognitive decline and dementia.^2^ Despite its importance there are few treatments that have been shown to delay disease progression in individuals with established disease.^3^ One reason for this is an incomplete understanding of the underlying pathophysiology, and therefore of the individual disease processes, which could be therapeutically targeted.

Recent evidence has suggested chronic neuroinflammation may play an important role in a variety of neurodegenerative diseases. Inflammation has also been proposed as a disease mechanism leading to both arterial and brain parenchymal injury in SVD.^4^ Circulating blood markers of inflammation and endothelial activation are elevated in SVD,^5^ and predict disease progression.^6^ Postmortem studies have reported evidence of central nervous system inflammation.^7^ A potential pathway by which hypoxia results in an inflammatory response and subsequent blood brain barrier breakdown and white matter damage has been proposed.^8^

Recent studies using ^11^C-PK11195 positron emission tomography (PET) to image neuroinflammation in patients with SVD have shown increased microglial activation in patients with SVD.^9^ Both increased whole brain activation and hot spots of increased activation in the white matter have been reported.^9,10^ This suggests that brain inflammation does occur in SVD.

However, it remains unclear whether this inflammation is playing a causal role in the disease process, or is merely occurring secondary to tissue damage. All PET studies to date have been cross-sectional, and whether increased central nervous system inflammation predicts future tissue damage and clinical events has not yet been determined. In this study, we performed follow-up magnetic resonance imaging (MRI) 1 year after a baseline ^11^C-PK11195 PET. We determined whether white matter regions destined to become new white matter hyperintensities (WMH) on the follow-up scan had evidence of altered inflammation in the baseline scans.

## Methods

The (anonymised) data that support the findings of this study are available from the corresponding author upon reasonable request by recognized researchers.

This case-control observational study was approved by the East of England – Cambridge South Ethics Committee (REC no: 16/EE/0468, IRAS project ID: 212632), and the Administration of Radioactive Substances Advisory Committee (ARSAC ref: 83/3886/35752). All participants provided written informed consent.

The subjects used in this study are the same as used in^10^; this article extends the analysis of these subjects. This study follows the STROBEreporting guideline (Strengthening the Reporting of Observational Studies in Epidemiology; Supplemental Material).^11^

### Subjects

Three groups of participants were recruited for this study, 2 with SVD (sporadic SVD, n=20; monogenic SVD cerebral autosomal dominant arteriopathy with subcortical infarcts and leukoencephalopathy [CADASIL], n=20) and 1 healthy control group (n=20).

For the sporadic SVD group, inclusion criteria were clinical evidence of lacunar stroke syndrome, with a corresponding lacunar infarct on diffusion weighted imaging at the time of stroke for cases initially imaged within 3 weeks of stroke, or an anatomically compatible lacunar infarct (≤1.5 cm diameter) on fluid attenuated inversion recovery (FLAIR)/T_1_ MRI for cases imaged later after stroke. In addition, to recruit a cohort with severe SVD, participants had to have confluent WMH defined as a score ≥2 on the Fazekas scale.^12^ Exclusion criteria were any cause of stroke other than SVD (eg, large artery stenosis >50% or a cardioembolic source) or a cortical infarct. Participants were recruited from an in-patient stroke service and out-patient stroke clinics based at Addenbrooke’s Hospital, Cambridge, UK.

The inclusion criteria for the CADASIL cohort was a confirmed genetic diagnosis of CADASIL, as defined by a typical cysteine changing *NOTCH3* mutation. Participants were recruited from a national CADASIL clinic based at Cambridge.

Participants in both SVD groups were not recruited until at least 3 months after a stroke to avoid changes secondary to acute injury, and in both groups mini mental state examination^13^ had to be >21 to ensure ability to consent and ensure subjects could comply with the protocol.

The healthy control group comprised 20 participants with no history of stroke or other major neurological disorder and were recruited from both the community and family/friends of patients. The age of the control subjects was chosen to cover the range of the 2 groups of SVD subjects. Later subjects were screened for age and sex to match those already recruited. No other patient details, other than those described above as inclusions/exclusions, were used to select subjects.

Subjects with contraindication to MRI were excluded. Females of childbearing potential were also excluded due to the administration of the radioactive ligand.

### PET/MRI Acquisition

All participants underwent PET and MRI on a GE SIGNA PET/MR scanner (GE Healthcare) at the Wolfson Brain Imaging Center in Cambridge, UK. Imaging comprised baseline simultaneous ^11^C-PK11195 PET and MRI, together with follow-up MRI at 1 year after the baseline imaging. The full baseline imaging protocol has been described previously.^10^

List-mode ^11^C-PK11195 PET data were acquired for 75 minutes following radioligand injection. The median injected ^11^C-PK11195 activity was 440 MBq (interquartile range, 401–483 MBq), with corresponding injected PK11195 mass values of 3.9 (interquartile range, 2.8–6.4) μg.

Simultaneously, whole brain noncontrast MRI was acquired using a 32-channel head coil (Nova Medical). The sequences included the following: (1) 3D T_1_-weighted fast-spoiled gradient echo sequence (brain volume [BRAVO]), flip angle=12°, inversion time=450 ms, field of view=28 mm, slice thickness=1 mm, number of slices=192, reconstructed matrix size=512×512; (2) axial susceptibility weighted imaging, flip angle=17°, repetition time=40.6 ms, echo time=24.2 ms, field of view=22 mm, slice thickness=2 mm, number of slices=70, reconstructed matrix size=256×256; (3) axial T_2_ FLAIR sequence, angled anterior commissure-posterior commissure, flip angle=160°, repetition time=8800 ms, echo time=120 ms, inversion time=2445 ms, field of view=22 mm, slice thickness=5 mm, number of slices=28, reconstructed matrix size=256×256; and (4) diffusion tensor imaging (DTI) acquired axially with the diffusion gradients applied in 63 directions with a b-value=1000 s/mm^2^; Echo time=minimum, repetition time=15763 ms, field of view=19.2 mm, slice thickness=2 mm, number of slices=65 to 70 depending on slice angulation (again images were aligned with the anterior commissure-posterior commissure line), reconstructed matrix size=256×256.

Follow-up imaging was performed at 1 year and included only the MRI protocol, which was identical to that described above.

### Magnetic Resonance Image Analysis

WMH were quantified across the whole brain on FLAIR images by a single trained rater using the semiautomatic contouring technique included in Jim analysis software version 7.0.5 (Xinapse Systems Limited, http://www.xinapse.com/j-im-7-software/). The baseline and follow-up FLAIR images were marked at the same time and presented to the rater in a random order. The rater was assessed to have an excellent intrarater reliability for repeat marking of the same scan with an ICC of 0.99 assessed from an independent training set of 10 scans marked with a gap of 7 to 14 days between attempts. This rater was not involved in the production of the PET data (described below) and was blinded to the PET results at the time of lesion marking. Following lesion marking, the follow-up FLAIR images were then registered to the baseline using rigid registration in the Advanced Normalization Tools (http://stnava.github.io/ANTs/) registration package,^14^ and the WMH masks were then transformed to this space.

**Figure 1.**
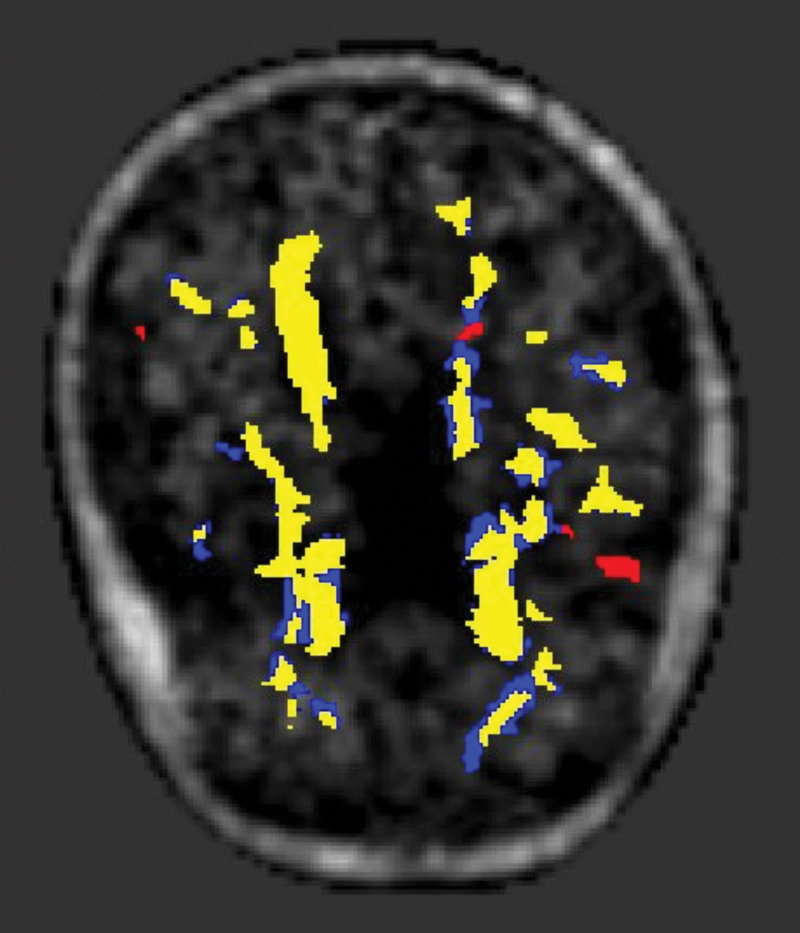
**An example image showing the registered lesions shown overlaid on the positron emission tomography (PET) binding map.** The yellow is the baseline lesions, the blue shows the unprocessed lesion growth, and the red shows the lesion growth after the dilation and minimum cluster size processes.

WMH growth was determined by subtraction of the registered follow-up and baseline WMH masks. To ensure that areas of WMH growth were not solely due to variation in WMH delineation, the baseline WMH masks were dilated by 2 voxels and this dilated mask was used to create a further WMH growth mask. In addition, this further WMH growth map was subjected to a minimum cluster size of 5 voxels to exclude isolated voxels, which may be due to WMH marking imprecision. These 2 definitions of new WMH voxels are noted as raw and processed voxels later in this work. An example of the WMH masks are shown in Figure 1. The reason for this is to minimize the number of baseline WMH voxels, or at least partial WMH voxels, in the new WMH masks. The dilation will exclude these partial volume voxels at the cost of also excluding some true new WMH voxels. The minimum cluster size removes odd voxels included due to variation in the marking process. These 2 definitions of new WMH voxels reflect inclusive and conservative definitions of WMH growth.

Lacunes were defined as cerebrospinal fluid-filled cavities at least 3 mm in diameter.^15^ They were manually delineated on the baseline FLAIR images by a single neurologist, blinded to subject identity, with both baseline T_1_ and FLAIR scans being visually inspected to confirm the presence of lacunes. The FLAIR image was registered to the T_1_ image using a rigid body transformation in Advanced Normalization Tools. The resulting transformation was used to resample the WMH mask from the FLAIR image to the T_1_ image using nearest-neighbor interpolation.

Each baseline T_1_ BRAVO image was processed using the segment routine in SPM12 (https://www.fil.ion.ucl.ac.uk/spm/software/spm12/). SPM segmentation provides tissue probability maps, and segments for each tissue class were determined as the voxels with >50% probability of belonging to that class, after removal of voxels in either the baseline or follow-up WMH masks. The tissue segments and WMH masks were used to create the normal appearing white matter (NAWM) and white matter (sum of NAWM and WMH) masks. These masks were then eroded by 3 mm using fslmaths (https://fsl.fmrib.ox.ac.uk/fsl/fslwiki), to effectively eliminate ventricular or gray matter contamination. It should be noted that the NAWM segment excludes WMH voxels from both baseline and follow-up.

DTI analysis was conducted on the baseline diffusion data as described previously.^16^ Briefly, FSL software (FDT; Oxford Center for Functional MRI of the Brain’s Diffusion Toolbox, http://fsl.fmrib.ox.ac.uk/fsl/fslwiki/FDT) was used for DTI preprocessing. Scans were corrected for eddy current effects and a binary brain mask in DTI space was determined. Fractional anisotropy (FA) and mean diffusivity (MD) maps were then calculated from this data. Voxels with MD values above 2.6×10^−^^4^ mm^2^/s were removed from analyses as these likely contain cerebrospinal fluid. Likewise, voxels with FA>1 were also removed. For each participant, the Oxford Center for Functional MRI of the Brain Linear Image Registration Tool was used to register the T1 weighted image to the B0 image, which could then be used to create a transformation of FLAIR to DTI. These registrations were all manually checked. Tissue segments and WMH masks were then transformed into DTI space. The tissue segments and WMH masks were then used to create tissue-specific diffusion parameter maps. The result of the registration process is shown in Figure 1. Whole WM DTI-based metrics have been shown to best predict cognitive decline in SVD and as such were chosen to investigate the health of the NAWM.^17,18^

### PET Analysis

List-mode PET data were histogrammed into 55 time frames and then reconstructed into images (128×128×89 matrix; 2.0×2.0×2.8 mm voxel size) using time-of-flight ordered subsets expectation maximization^19^ with 16 subsets, 6 iterations, and no smoothing. Attenuation correction included the use of a multisubject atlas method^20^ and improvements to the MRI brain coil component.^21^ Image reconstruction also included corrections for random coincidences, dead time, normalization, scattered coincidences, radioactive decay, and sensitivity.

SPM12 (https://www.fil.ion.ucl.ac.uk/spm/software/spm12/) was used to realign each dynamic image series and hence ameliorate the impact of head motion. A mean realigned PET image was then used to co-register each realigned dynamic PET image series to the T_1_ BRAVO magnetic resonance image from the same scan.

To estimate specific binding of ^11^C-PK11195, binding potential relative to a nondisplaceable compartment (BP_ND_) was determined with a basis function version of the simplified reference tissue model incorporating correction for vascular binding.^22^ TSPO is expressed by cell types other than microglia, including endothelial cells. To account for this, correction of the PET signal for binding to vascular endothelial cells has been developed,^23^ and this was applied in the reference tissue kinetic model used in this work to increase the specificity for TSPO expressed in parenchymal brain tissue. The white matter reference tissue input was estimated with supervised cluster analysis^22^ using library data determined from control scans of another ^11^C-PK11195 project on the same scanner with the same acquisition and processing protocol. A 4 mm full-width at half-maximum Gaussian was applied to the dynamic images prior to production of BP_ND_ maps using simplified reference tissue model.

As described in^10^ we determined hot spots of inflammation at baseline by defining these as voxels with a binding value >95 centile of values from the white matter in the control subjects.

### Data and Statistical Analysis

#### Lesion Growth

To test whether there was lesion growth in each group, the Wilcoxon signed-rank test was used on the baseline and follow-up lesion volumes. Lesion growth was compared between the groups using the Mann-Whitney *U* test. These tests were used due to nonnormal distributions for this variable.

#### Baseline Microglial Binding of Voxels That Become New WMH at 1 Year

To investigate this, the WMH and WMH growth masks were transformed from FLAIR space to PET space via the native T_1_ BRAVO and the T_1_ BRAVO aligned to the PET images by concatenating the transformations described above. This allowed estimates of ^11^C-PK11195 BP_ND_ to be obtained in baseline WMH and NAWM, and WMH growth voxels. These values were then compared using the Student paired *t* test for the whole population and the individual subject groups.

#### Baseline Diffusion Characteristics of Voxels That Become New WMH at 1 Year

The FLAIR and BRAVO images were also registered to the DTI B0 data using Advanced Normalization Tools allowing baseline MD and FA to be determined in baseline WMH and NAWM, and WMH lesion growth voxels. The MD and FA values were then compared using the Student paired *t* test for the whole population and the individual subject groups.

#### Fate of Hot Spots of Inflammation Seen at Baseline

The percentage of hot spot voxels that become WMH was determined along with the percentage of white matter voxels that become new WMH, these values were compared in the sample as a whole and each subject group using the paired samples *t* test.

All variables in the above analyses, apart from lesion growth, were normally distributed.

## Results

PET data were successfully collected from 17 control, 16 sporadic SVD, and 14 CADASIL participants; for the 13 of 60 recruited subjects in which a PET data set was not collected and this was due to ligand production failure (4 subjects), ligand QC failure (4 subjects), or low production yield (5 subjects). In one further case, MR imaging failed due to a scanner issue and this subject was also excluded leaving 13 in the CADASIL group. There was no difference in the lesion load between subjects with and without imaging, using the independent samples *t* test *P* values were 0.99, 0.13, and 0.93 for the control SVD and CADASIL groups, respectively. Baseline subject demographic and clinical data are shown in Table 1. Follow-up MRI occurred at (mean±SD) 381±25 days, range 358 to 485 days.

**Table 1.**
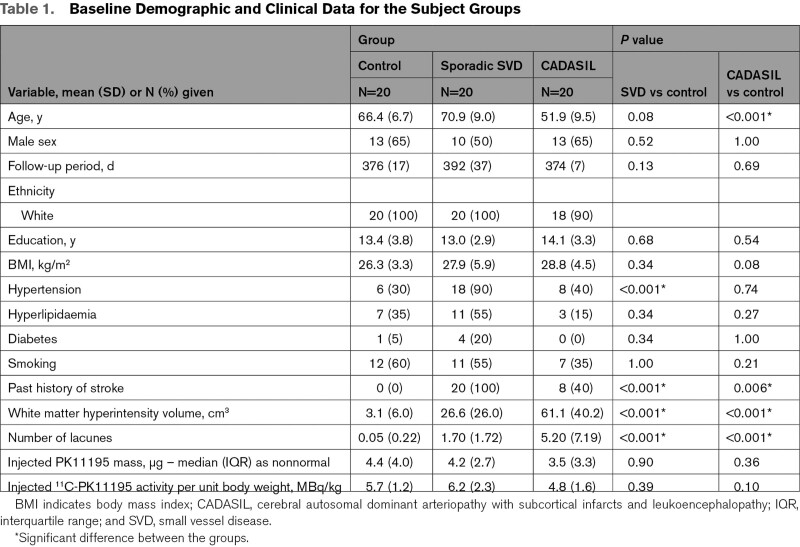
Baseline Demographic and Clinical Data for the Subject Groups

### Baseline Microglial Binding Results

These results have been presented previously,^10^ but are of relevance so are summarized here. There was an increase in the volume of hot spots of increased ^11^C-PK11195 BP_ND_ in the NAWM (*P*=0.003) and WMH (*P*=0.004) of SVD subjects compared to controls. There was no significant difference in the mean ^11^C-PK11195 BP_ND_ seen between the patient groups in either NAWM or WMH, but it was lower in WMH than in NAWM for both the SVD and CADASIL groups. In addition, ^11^C-PK11195 BP_ND_ was lower in the new WMH tissue surrounding the baseline WMH than in NAWM (*P*<0.001 for all 3 groups).

### WMH Lesion Growth

**Figure 2.**
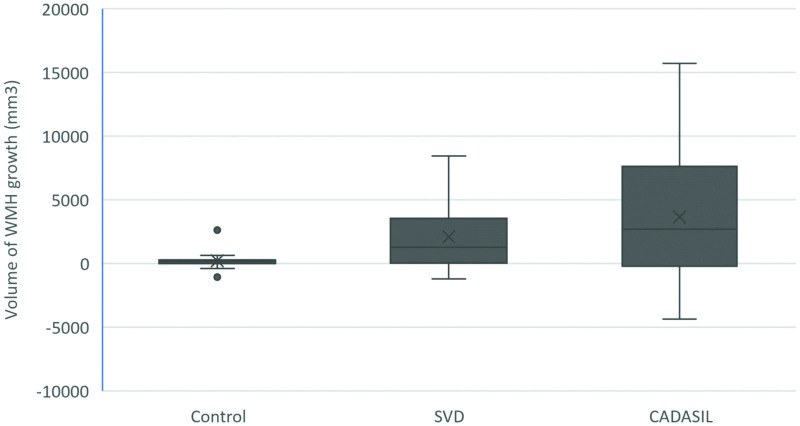
**Volume of WMH growth over 1 y in the 3 subject groups.** CADASIL indicates cerebral autosomal dominant arteriopathy with subcortical infarcts and leukoencephalopathy; SVD, small vessel disease; and WMH, white matter hyperintensities.

The volume of WMH growth over 1 year for the 3 subject groups is shown in Figure 2.

The WMH growth (prior to dilation and cluster limits) in the 3 groups was 0.2±0.7 mL for the control group, 2.1±2.6 mL for the sporadic SVD group, and 3.6±5.2 mL for the CADASIL group. The Wilcoxon-signed ranks test showed that there was significant growth in the population as a whole (*P*=0.001) and in the sporadic SVD and CADAIL groups (*P*=0.002 and *P*=0.008, respectively), but not for the control group (*P*=0.142), and the Mann-Whitney *U* test showed significant differences in lesion growth between the control group and both the SVD (*P*=0.027) and CADASIL (*P*=0.014) groups. There was no statistical difference between the SVD and CADASIL groups (*P*=0.372).

#### Baseline Microglial Binding of Voxels That Become New WMH at 1 Year

**Figure 3.**
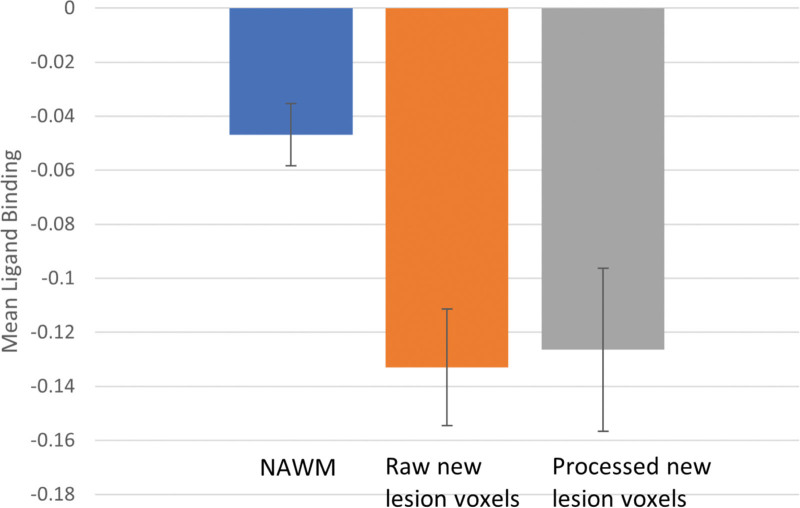
**Mean ^11^C-PK11195 binding potential relative to a nondisplaceable compartment (BP_ND_) in the normal appearing white matter (NAWM), raw new white matter hyperintensities (WMH) voxels, and the processed new WMH voxels for the whole sample.** Error bars denote the 95% CI.

Figure 3 shows mean ^11^C-PK11195 BP_ND_ for voxels destined to become new WMH, for both raw and processed definitions of new lesion voxels, and for the rest of the NAWM. Across the whole dataset, there was lower ^11^C-PK11195 binding in voxels destined to become new WMH for both definitions.

When looking at the whole population, the difference in ^11^C-PK11195 BP_ND_ between new WMH voxels and NAWM was statistically significant (*P*<0.001) for both definitions of new WMH voxels. When the 3 subject groups were analysed separately, the control group showed significant differences (*P*=0.028 and *P*=0.005 for the raw and processed new lesion voxels, respectively) as did the SVD group (*P*=0.019 and *P*=0.004); the results for the CADASIL group were less marked with *P* values of *P*=0.006 and *P*=0.22 for the 2 WMH lesion growth definitions.

There was no difference in ^11^C-PK11195 BP_ND_ between the new WMH voxels and existing WMH for the whole population (*P*=0.08 and *P*=0.21 for the raw and processed new lesion voxels). When the 3 groups were analyzed separately, the control and SVD groups showed no difference (*P*>0.39); however, for the CADASIL group there was significantly higher ^11^C-PK11195 BP_ND_ in the new WMH voxels compared to the extant WMH (−0.13 versus −0.16, *P*=0.046 and −0.08 versus −0.16, *P*=0.001 for the raw and processed new lesion voxels, respectively).

#### Baseline Diffusion Characteristics of Voxels That Become New WMH at 1 Year

Across the whole dataset, mean MD was higher and mean FA was lower in new WMH voxels than in NAWM (all *P*<0.001; Tables 2 and 3) consistent with new WMH showing tissue damage on DTI, years prior to developing into new WMH.

**Table 2.**
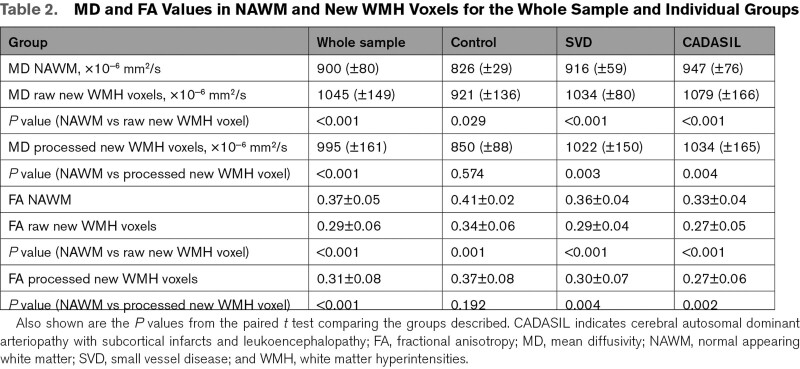
MD and FA Values in NAWM and New WMH Voxels for the Whole Sample and Individual Groups

**Table 3.**
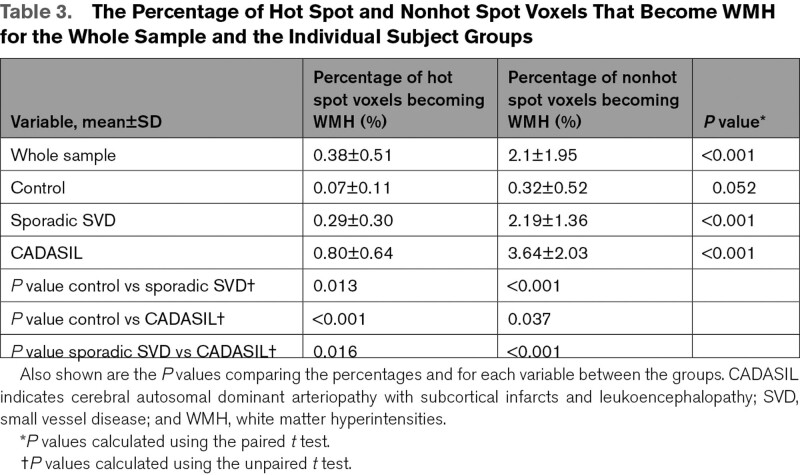
The Percentage of Hot Spot and Nonhot Spot Voxels That Become WMH for the Whole Sample and the Individual Subject Groups

When looking at the individual groups, in both the sporadic SVD and CADASIL groups, similar findings were observed with higher MD and lower FA in the new WMH voxels than NAWM (Table 2). In the control group, a similar significant difference was found for the raw new WMH voxels but not for the processed voxels (Table 2).

#### Fate of Hot Spots of Inflammation Seen at Baseline

The percentage of hot spot and nonhot spot voxels that become WMH are shown in Table 3. There were significant differences in the percentage of hot spot voxels and nonhot spot voxels that became WMH for the whole sample (*P*<0.001) and the SVD and CADASIL group (*P*<0.001), while the control group showed a borderline difference (*P*=0.052) in all cases with a higher percentage of nonhot spot voxels converting to WMH than hot spot voxels.

When comparing the percentage of hot spot voxels that become WMH between groups, there were significant differences between the 3 groups (*P*<0.016) and the same was true for the nonhot spot voxels (*P*<0.037), in both cases the CADASIL had the highest percentage of voxels convert and the controls the least.

## Discussion

Inflammation has been associated with SVD pathology for a long time, including inflammation in the walls and tissue surrounding perforating arterioles and microglial activation. Recent studies have demonstrated the presence of central nervous system inflammation in patients with SVD, including evidence of microglial activation. Both globally increased ^11^C-PK11195 binding and focal hot spots of ^11^C-PK11195 binding in the white matter have been described.^9,10^ However, the relationship of this inflammation to disease progression remains undetermined. Our results demonstrate that white matter tissue destined to develop into new WMH over the subsequent year is associated with lower neuroinflammationthan surrounding NAWM at baseline. Where, in this context, neuroinflammation means microglial activation. Furthermore, such tissue destined to develop into WMH is characterized by white matter ultrastructural damage as evidenced on DTI at baseline.

Whether central nervous system inflammation represents a causal process in tissue damage secondary to SVD, or whether it is merely a secondary consequence, remains uncertain. It is also uncertain which aspects of inflammation may play a part in the relationship. Our results suggest that if it is indeed causal then the microglial activation aspect of inflammation is likely to play a role at an earlier stage in the development of WMH, and by a year prior to the WMH becoming visible the tissue is already irreversibly damaged. This has implications both in determining the time course of inflammation and tissue damage in SVD, and also in intervening with anti-inflammatory therapies to reduce WMH progression. This implies that such interventions would need to be performed at an earlier stage in the disease.

In previous analysis of the baseline data from the same dataset, we showed hot spots of increased ^11^C-PK11195 binding in the white matter, often adjacent to white matter lesions.^10^ This raises the possibility that inflammation may be one of the mechanisms by which WMH grow. This is consistent with data from an animal model^8^ of white matter ischemia in which chronic hypoperfusion resulted in hypoxia with HIF-1 alpha production and an inflammatory response, which led to subsequent matrix metalloproteinase 9 production, blood brain barrier breakdown, and tissue damage. White matter damage was reduced by treatment with minocycline, which has known anti-inflammatory and blood brain barrier stabilizing properties, suggesting that the pathway was indeed causal.^8^ However, the lack of hot spot to WMH conversion adds support for the idea that this is a long-term process and if there is a link in the process that increased microglial activation occurs long before WMH transformation.

Although the absolute values of the percentage of voxels becoming WMH differs between the groups, this just reflects the different rate of lesion growth in the groups and the behavior of the 3 groups is similar in terms of the relationship between the hot spot and nonhot spot voxels. Although the animal model work raises the exciting possibility that therapies targeting the inflammatory response could reduce white matter damage in SVD. Our results emphasise that more understanding of the time course of inflammation and white matter damage is required before an appropriate intervention study could be planned.

In areas destined to become new WMH, we found not only reduced inflammation but also decreased FA and increased MD. This pattern of diffusion abnormalities is indicative of white matter ultrastructural damage, and is seen in many white matter diseases including SVD. It suggests these areas determined to become WMH are already abnormal at baseline.^24^

We chose DTI-based metrics as the MRI marker of damage to the tissue rather than magnetisation transfer imaging, or other measure of the neuropil as they have been shown to best predict cognitive decline in SVD.^17,18^ Estimating damage to WM using DTI allows assessment of the microstructure of the tissue, which reflects the ability of the tissue to transmit signals across the brain.

This work suggests changes to microglial activation in WMH-destined voxels; however. it is not clear why this would be the case. The lower binding potential could represent lower binding site density in the tissue compared to the reference tissue or an over-correction for binding to vascular endothelium. Values would also be affected by changes in nonspecific binding, such as to astrocytes, so processes occurring in these tissues other than microglial activation may have an impact on the binding potential seen.

We have not investigated other elements of inflammation in this study, so are unable to comment on whether these are related to lesion formation.

Our study has a number of strengths. It is the first study to look longitudinally at the relationship between neuroinflammation, measured using PET microglial activation, and white matter lesion progression in vivo in man. We studied both sporadic SVD, for which the major risk factor is hypertension, and also a monogenic form of SVD, CADASIL in which the small vessels are damaged by a different molecular process, namely mutations in the *NOTCH3* gene. However, it also has limitations. First, we studied only 2 timepoints, which does not allow complete understanding of the relationship between the time course of inflammation and new lesion formation. Second, the PET imaging has lower spatial resolution compared with the MRI imaging meaning that the binding potential measurements are subject to greater partial volume error, particularly with regard to small areas of lesion growth. Third, due to reliability issues with radiotracer production, not all patients were able to have PET scans, this, coupled with the relatively small size of the study in total and in each group, does reduce the power of the study. The moderate power may have reduced associations, particularly in the control group in whom there was relatively little new white matter lesion growth. Fourth, the data showed that there was no significant lesion growth in the control group, which means that the results from this group are only of limited value; however, there was growth in the other 2 groups. Lastly, it should be noted that the groups were not perfectly matched. As the age range of sporadic SVD and CADASIL subjects differs, there was increased hypertension in the SVD group. The MR variables WMH volume and number of lacunes also differ between the groups due to the presence of disease. These differences may have an impact on the results seen, however are difficult to match due to the characteristics of the disease.

We have not corrected any *P* values for multiple comparisons, most significant results would survive correction; however, the weaker differences would disappear. This should be borne in mind when interpreting the results.

In conclusion, this work has shown that there is low microglial activity in voxels destined to become lesion up to 1 year later. This tissue has diffusion parameter abnormalities, which suggest that the tissue is already damaged at baseline and already on the pathway to lesion.

## Article Information

### Acknowledgments

The authors acknowledge the help of the radiographers at the Wolfson Brain Imaging Center, University of Cambridge, in scanning the subjects.

### Sources of Funding

Recruitment was supported by the National Institute for Health Research Clinical Research Network. The study was funded by a Medical Research Council (MRC) experimental medicine grant (MR/N026896/1). Professor Markus is supported by a National Institute of Health Research (NIHR) Senior Investigator award. This work was supported by infrastructural support from the Cambridge BHF Center of Research Excellence [RE/18/1/34212]. Dr Brown is supported by an Association of British Neurologists Clinical Research Training Fellowship funded by the Guarantors of Brain. The research and Professor O’Brien is supported by the NIHR Cambridge Biomedical Research Center (BRC-1215-20014). The views expressed are those of the author(s) and not necessarily those of the NIHR or the Department of Health and Social Care.

### Disclosures

Dr Tozer was funded for this work as both salary and grant support by the Medical Research Council (MRC). Professor O’Brien discloses grants from the MRC, Alliance Medical, Eli Lilley and Company and Merck. He also holds consultancy roles for Biogen, GE Healthcare, Novo Nordisk, Roche and TauRx Therapeutics. Professor Markus discloses grant support from the British Heart Foundation. The other authors report no conflicts.

## Supplementary Material
