# Supplementary material for: Do Regions of Increased Inflammation Progress to New White Matter Hyperintensities?: A Longitudinal Positron Emission Tomography-Magnetic Resonance Imaging Study
Source: Stroke. 2023 Jan 9;54(2):549–57. doi: 10.1161/STROKEAHA.122.039517 (PMC9855729; doi:10.1161/STROKEAHA.122.039517)
Supplement: Supplementary file 1 [file str-54-549-s001.pdf]

STROBE Statement—checklist of items that should be included in reports of observational studies

	Item No.	Recommendation	Page No.	Relevant text from manuscript
**Title and abstract**	1	(*a*) Indicate the study's design with a commonly used term in the title or the abstract	Abstract	Forty subjects with SVD (20 sporadic, and 20 CADASIL), and 20 controls were imaged at baseline with both ^{11}C-PK11195 PET and MRI.
		(*b*) Provide in the abstract an informative and balanced summary of what was done and what was found	Abstract	See manuscript
**Introduction**				
Background/rationale	2	Explain the scientific background and rationale for the investigation being reported	2	See manuscript
Objectives	3	State specific objectives, including any prespecified hypotheses	4	We determined whether white matter regions destined to become new white matter hyperintensities on the follow-up scan, had evidence of altered inflammation in the baseline scans.
**Methods**				
Study design	4	Present key elements of study design early in the paper	4-5	See manuscript
Setting	5	Describe the setting, locations, and relevant dates, including periods of recruitment, exposure, follow-up, and data collection	4	Participants were recruited from an in-patient stroke service and out-patient stroke clinics based at Addenbrooke's Hospital, Cambridge, UK.<br>Participants were recruited from a national CADASIL clinic based at Cambridge.
Participants	6	(*a*) *Cohort study*—Give the eligibility criteria, and the sources and methods of selection of participants. Describe methods of follow-up<br>*Case-control study*—Give the eligibility criteria, and the sources and methods of case ascertainment and control selection. Give the rationale for the choice of cases and controls<br>*Cross-sectional study*—Give the eligibility criteria, and the sources and methods of selection of participants	4	For the sporadic SVD group inclusion criteria were:<br>Clinical evidence of lacunar stroke<br>Confluent white matter hyperintensities defined as a score ≥2 on the Fazekas scale<br>Exclusion criteria:<br>Any cause of stroke other than

				SVD or a cortical infarct<br>Inclusion criteria for the CADASIL cohort:<br>Confirmed genetic diagnosis of CADASIL<br>Participants not recruited until at least 3 months after a stroke<br>Mini mental state examination had to be >21.
		(*b*) *Cohort study*—For matched studies, give matching criteria and number of exposed and unexposed<br>*Case-control study*—For matched studies, give matching criteria and the number of controls per case	9	Table 1 and PET data was collected from 17 control, 16 sporadic SVD and 14 CADASIL participants; There was no difference in the lesion load between subjects with and without imaging, using the independent samples t-test p values were 0.99, 0.13 and 0.93 for the control SVD and CADASIL groups. Baseline subject demographic and clinical data are shown in Table 1. Follow-up MRI occurred at (mean±SD) 381±25 days, range 358-485 days.
Variables	7	Clearly define all outcomes, exposures, predictors, potential confounders, and effect modifiers. Give diagnostic criteria, if applicable	6-9	See manuscript
Data sources/ measurement	8*	For each variable of interest, give sources of data and details of methods of assessment (measurement). Describe comparability of assessment methods if there is more than one group	6-9	See manuscript
Bias	9	Describe any efforts to address potential sources of bias	NA	
Study size	10	Explain how the study size was arrived at	NA	

Continued on next page

Quantitative variables	11	Explain how quantitative variables were handled in the analyses. If applicable, describe which groupings were chosen and why	6-9	See manuscript
Statistical methods	12	(*a*) Describe all statistical methods, including those used to control for confounding	8-9	See manuscript
		(*b*) Describe any methods used to examine subgroups and interactions	NA	
		(*c*) Explain how missing data were addressed	9	Subjects with missing data were excluded from the relevant comparison
		(*d*) *Cohort study*—If applicable, explain how loss to follow-up was addressed<br>*Case-control study*—If applicable, explain how matching of cases and controls was addressed<br>*Cross-sectional study*—If applicable, describe analytical methods taking account of sampling strategy	9	Loss to follow up was addressed by removing the subject from the relevant comparisons
		(*e*) Describe any sensitivity analyses	NA	
**Results**				
Participants	13*	(a) Report numbers of individuals at each stage of study—eg numbers potentially eligible, examined for eligibility, confirmed eligible, included in the study, completing follow-up, and analysed	9	PET data was successfully collected from 17 control, 16 sporadic SVD and 14 CADASIL participants. In one further case MR imaging failed due to a scanner issue and this subject was also excluded leaving 13 in the CADASIL group.
		(b) Give reasons for non-participation at each stage	10	See above
		(c) Consider use of a flow diagram		
Descriptive data	14*	(a) Give characteristics of study participants (eg demographic, clinical, social) and information on exposures and potential confounders	Table 1	Table 1
		(b) Indicate number of participants with missing data for each variable of interest	NA	
		(c) *Cohort study*—Summarise follow-up time (eg, average and total amount)	9	Follow-up MRI occurred at (mean±SD) 381±25 days, range 358-485 days
Outcome data	15*	*Cohort study*—Report numbers of outcome events or summary measures over time	10-11	See manuscript
		Case-control study—Report numbers in each exposure category, or summary measures of exposure		
		Cross-sectional study—Report numbers of outcome events or summary measures		
Main results	16	(*a*) Give unadjusted estimates and, if applicable, confounder-adjusted estimates and their precision (eg, 95% confidence interval). Make clear which confounders were adjusted for and why they were included	Tables 2 and 3	See manuscript
		(*b*) Report category boundaries when continuous variables were categorized	NA	
		(*c*) If relevant, consider translating estimates of relative risk into absolute risk for a meaningful time period	NA	

Continued on next page

Other analyses	17	Report other analyses done—eg analyses of subgroups and interactions, and sensitivity analyses	NA	
**Discussion**				
Key results	18	Summarise key results with reference to study objectives	11	Our results demonstrate that white matter tissue destined to develop into new WMH over the subsequent year, is associated with lower "neuroinflammation" than surrounding normal appearing white matter at baseline. Furthermore, such tissue destined to develop into WMH is characterised by white matter ultrastructural damage as evidenced on DTI at baseline.
Limitations	19	Discuss limitations of the study, taking into account sources of potential bias or imprecision. Discuss both direction and magnitude of any potential bias	13	Firstly, we studied only two timepoints which does not allow complete understanding of the relationship between the time course of inflammation and new lesion formation. Secondly the PET imaging has lower spatial resolution than the MRI meaning that the binding potential measurements are subject to greater partial volume error. Thirdly due to reliability issues with radiotracer production not all patients were able to have PET scans, thereby reducing the power. Lastly the moderate power may have reduced associations, particularly in the control group in whom there was relatively little new white matter lesion growth.
Interpretation	20	Give a cautious overall interpretation of results considering objectives, limitations, multiplicity of analyses, results from similar studies, and other relevant evidence	12-13	See manuscript
Generalisability	21	Discuss the generalisability (external validity) of the study results	12	This work suggests changes to microglial activation in WMH destined voxels. The lower binding potential could represent lower binding site density

				in the tissue compared to the reference tissue and/or an over-correction for binding to vascular endothelium.
**Other information**				
Funding	22	Give the source of funding and the role of the funders for the present study and, if applicable, for the original study on which the present article is based	13	Recruitment was supported by the National Institute for Health Research Clinical Research Network. The study was funded by a Medical Research Council (MRC) experimental medicine grant (MR/N026896/1). HSM is supported by a National Institute of Health Research (NIHR) Senior Investigator award. This work was supported by infrastructural support from the Cambridge BHF Centre of Research Excellence [RE/18/1/34212]. RBB is supported by an Association of British Neurologists Clinical Research Training Fellowship funded by the Guarantors of Brain. The research and JOB is supported by the NIHR Cambridge Biomedical Research Centre (BRC-1215-20014). The views expressed are those of the author(s) and not necessarily those of the NIHR or the Department of Health and Social Care.

*Give information separately for cases and controls in case-control studies and, if applicable, for exposed and unexposed groups in cohort and cross-sectional studies.

Note: An Explanation and Elaboration article discusses each checklist item and gives methodological background and published examples of transparent reporting. The STROBE checklist is best used in conjunction with this article (freely available on the Web sites of PLoS Medicine at http://www.plosmedicine.org/, Annals of Internal Medicine at http://www.annals.org/, and Epidemiology at http://www.epidem.com/). Information on the STROBE Initiative is available at www.strobe-statement.org.
